# Tracking of Blood Pressure Among Adolescents and Young Adults in an Urban Slum of Puducherry

**DOI:** 10.4103/0970-0218.40879

**Published:** 2008-04

**Authors:** MB Soudarssanane, S Mathanraj, MM Sumanth, Ajit Sahai, M Karthigeyan

**Affiliations:** Department of Preventive and Social Medicine, JIPMER, Puducherry, India

**Keywords:** Adolescents/young adults, cohort study, incidence of hypertension, risk factors, tracking of blood pressure

## Abstract

**Background:**

Early diagnosis of hypertension (HT) is an important strategy in its control. Tracking of blood pressure (BP) has been found useful in identifying persons with potential HT, particularly in youngsters. A cohort of 756 subjects (with baseline information as a cross-sectional study in 2002) was followed up in 2006 to comment on the distribution of BP and its attributes.

**Objectives:**

To track BP distribution in a cohort of adolescents and young adults, and assess the persistence of high/low normotensives; to measure the incidence of HT and study the relationship of BP with age, sex, socioeconomic status, BMI, physical exercise, salt intake, smoking and alcohol consumption.

**Materials and Methods:**

The baseline study cohort (2002) of 756 subjects (19-24 years) in urban field area of Department of Preventive and Social Medicine, JIPMER, was followed up between May and November 2006 by house visits for measurement of sociodemographic variables, anthropometry, salt intake, physical activity and BP.

**Results:**

A total of 555 subjects from the 2002 cohort were contacted (73.4%), in that 54.5% subjects who were below 5^th^ percentile, 93.6% subjects between 5^th^ and 95^th^ percentiles and 72% of those above 95^th^ percentile previously persisted in the same cut-offs for systolic blood pressure (SBP). The corresponding figures for diastolic blood pressure (DBP) were 46.2, 92.2 and 74.1%, respectively. Shift from one cut-off to another was not significant for both SBP and DBP, proving the tracking phenomenon. Annual incidence of HT was 9.8/1000. Baseline BP was the significant predictor of current BP for the entire cohort; BMI and salt intake were significant predictors only in certain sections of the study cohort.

**Conclusions:**

Early diagnosis of hypertension even among adolescents/young adults is an important preventive measure, as tracking exists in the population.

Coronary risk factors such as hypertension, smoking, physical inactivity, obesity and improper diet are fairly widespread.(1) Cardiovascular diseases, particularly hypertension, account for high mortality in the form of cardiovascular strokes in countries like India, Taiwan and Japan.(2) In Indian adolescent school children, there is a high prevalence of obesity, hypertension, hypercholesterolemia and high fat diet.(3) Studies(4,5) from Boston and Pennsylvania had commented that the role of hypertension as a risk factor is clear, and familial aggregation of blood pressure and tracking phenomenon support the concept that children with hypertension are likely to be hypertensive as adults and will be at risk for early CHD. In Puducherry, several studies on distribution of blood pressure and prevalence of hypertension (including its determinants) have been conducted in 1991, 1996 (among adults) and 2002(6) among adolescents. As a follow up of the latest study on adolescents, this work was undertaken with the following objectives:

1. To track BP distribution among the cohort of adolescents/young adults (of the earlier 2002 study) and assess if persons who were high normal/low normal/hypertensive continue to have similar trends.
2. To assess the incidence of hypertension in the study cohort.
3. To asses the relationship between BP and hypertension in the study cohort with certain factors like age, sex, education, occupation, income, BMI, physical exercise, salt intake, smoking and alcohol consumption.

## Materials and Methods

This cohort study was carried out in an urban field practice area of Department of Preventive and Social Medicine, Jawaharlal Institute of Post-graduate Medical Education and Research (JIPMER), Puducherry. The study population was the same 756 participants of the 2002 study (now 19-24 years), covering adolescents and young adults. Informed consent was taken from every subject in the local language (Tamil). Data collection was by house visits with a pre-tested questionnaire using the same definitions/parameters as for the reference study, for the following variables: (1) identification data: age, sex, religion, social class [Kuppuswamy's scale(7) - classified as social class I to V], (2) physical activity: very good [physical exercise like manual work per se or sports activity or other physical exercise for >3 h/day], moderate [1-3 h/day], mild [<1 h/day], sedentary [nil physical activity], (3) per capita salt intake, (4) history of parental diabetes mellitus/hypertension, (5) smoking: light [< 5 cigarettes/day], moderate [6-10 cigarettes/day], heavy [>10 cigarettes/day], non-smoker [never smoked], (6) alcohol consumption: occasional [once or twice a month], frequent [once or twice a week], always [>twice a week], never consumed, (7) anthropometry: Height - recorded to nearest 0.1 cm with stadiometer, weight to nearest 100 g with solar weighing machine, (8) measurement of BP: the conditions followed for measuring BP were as described by Dasgupta.(8) The subject was asked to rest for 5-10 min if he/she had engaged in physical activity. The WHO criteria(9) were followed in recording the BP and the average of two readings recorded 3 min apart was taken as BP. Systolic BP more than 160 mmHg and/or DBP more than 100 mmHg was severe hypertension, SBP 140-159 mmHg and/or DBP 90-99 mmHg was moderate hypertension. For adolescents, values more than 95^th^ percentile of BP were taken as hypertension. Analysis was done with Statistical Package for Social Studies (SPSS) version 13.0 using comparison of means, McNemar test, marginal homogeneity, correlation, multiple regression and paired ‘*t*’ test.

## Results

Out of 756 members of the earlier cohort, 555 (73.41%) were studied (292 males, 263 females). Of the 201 lost to follow-up, 15 had shifted residence, 15 girls got married and resettled, 4 had died and the remaining 167 could not be contacted even after four visits. The follow-up percentage varied from 77.7% to 66.3% for individual age groups. Age-wise composition of the 555 subjects studied was almost same as the 201 lost to follow-up, as available from the baseline cohort of 2002 (*P* = 0.109) [Table 1]

**Table 1:** Distribution of reference cohort and follow-up cohort by age and gender

Reference cohort (2002)				Study cohort (2006)							
	
				Coverage*				Non-coverage*			
		
Age	M	F	T	M	F	T	%	M	F	T	%
15	81	64	145	58	50	108	74.5	22	14	37	25.5
16	57	69	126	44	52	96	76.1	13	17	30	23.9
17	56	56	112	47	40	87	77.6	9	16	25	22.4
18	77	71	148	62	53	115	77.7	14	18	33	22.3
19	80	62	142	53	41	94	66.7	27	20	48	33.4
20	40	43	83	28	27	55	66.3	11	16	28	33.7
Total	391	365	756	292	263	555	73.4	96	101	197†	26.6

M = male, F = female, T = total;

* *P* = 0.109;

† Four had died

## BP distribution in study cohort

Compared with the reference 2002 cohort, in the present 2006 cohort, there was persistence of subjects in high/low/normal ranges of BP. The 5^th^ and 95^th^ percentiles for systolic blood pressure (SBP) for the age of 19 were 100 mmHg and 133.55 mmHg, and for diastolic blood pressure (DBP) 66 mmHg and 90 mmHg respectively. At 24 years of age, the 5^th^ and 95^th^ percentiles for SBP were 106 mmHg and 140 mmHg respectively and corresponding DBP values were 67.6 mmHg and 94 mmHg. The overall 5^th^ and 95^th^ percentiles (for all ages) for SBP were 104 mmHg and 132.20 mmHg. Corresponding values for DBP were 66 mmHg and 90.0 mmHg

## Tracking of BP and incidence of hypertension

Comparing the SBP of the study and reference cohorts, persons in extreme percentiles in 2002 continued in respective categories (below 5^th^ percentile, 5^th^ to 95^th^ percentiles and above 95^th^ percentile). The shift from one cut-off to another was not significant. Of those in 5^th^ to 95^th^ percentile of reference cohort, 93.6% continued within the same cut-offs. More than 70% of individuals of reference cohort who were in >95^th^ percentile continued to be so in study cohort. Similarly, for DBP, 92.2% individuals of reference cohort remained in 5^th^ to 95^th^ percentile. In the extreme categories (<5^th^ percentile and >95^th^ percentile) 74.1% of subjects continued in the same. The shift from one cut-off to another was not statistically significant [Table 2]. Similarly the shift from one cut-off to another was not significant at various levels like deciles, quintiles, quartiles and tertiles. Thus, tracking is demonstrated to be significant, viz., high/low normotensives tend to persist in their respective percentiles - thereby enabling early identification.

**Table 2:** Comparisons of blood pressure and hypertension in the reference and study cohorts

SBP new percentiles (2006)		SBP old percentiles (2002)			
		
		<5^th^percentile	5^th^ to 95^th^ percentile	>95^th^ percentile	Total
	<5^th^ percentile	18 (54.5%)	17(3.4%)	0	35
	5^th^ to 95^th^ percentile	15 (45.5%)	465 (93.6%)	7 (28.0%)	487
	>95^th^ percentile	0	15(3.0%)	18(72.0%)	33
	Total	33	497	25	555
MH statistic = 49, *P*-value = 0.414 (Marginal homogeneity test)					
DBP new percentiles (2006)		DBP old percentiles (2002)			
		
		<5^th^ percentile	5^th^ to 95^th^ percentile	>95^th^ percentile	Total
	<5^th^ percentile	12 (46.2%)	19(3.8%)	0	31
	5^th^ to 95^th^ percentile	14 (53.8%)	463 (92.2%)	7 (25.9%)	484
	>95^th^ oercentile	0	20 (4.0%)	20(74.1%)	40
MH statistic = 53, *P*-value = 0.16 (Marginal homogeneity test)					
Hypertensives (2006)		Hypertensives (2002)			Total
		
		Normotensive	Hypertensive		
	Normotensive	489 (95.9%)	15(32.6%)		504
	Hypertensive	20(4.1%)	31 (67.4%)		51
	Total	509	46		555
McNemar statistic: 2.6, *P*-value = 0.398 (McNemar test)					

Of the earlier 46 hypertensives, 31 (67%) continued to be so. Of the 509 normotensives, 489 (96%) continued in same range with 20 new occurrences of hypertension; that is 39.2/1000 over 4 years. Hence for 1 year the incidence was 9.8/1000 (5/555). Among 292 males and 263 females, 42 and 9 had hypertension giving incidences of 13.4 and 6.02/1000 respectively (*P* = 0.005) [Table 3]. Incidences among the different age groups (19-24) were 2.5, 5.5, 3.3, 9.2, 14.7 and 34.2/1000, respectively. All the 20 new hypertensives were isolated diastolic hypertension (IDH).

**Table 3:** Incidence/prevalence of hypertension among the study cohort of adolescents and young adults

Variable	Prevalence		Incidence	
		
	Number	Per 1000	Number	Per 1000
Overall	51	92	20	9.8
Gender				
Male	42	143	14	13.4
Female	9	34	6	6.0	
Parental h/o HT				
Paternal	4	121	2	21.2
Maternal	8	195	3	17.5
Both parents	3	333	1	50.0
Anthropometry				
Obese	9	288	2	12.3
Normal BMI	42	71	18	13.5
Salt intake				
<10g/d	4	26	2	2.0
>10g/d	47	176	18	16.5
Physical activity				
Mild activity	16	91	7	7.0
Moderate	31	126	7	11.0
Very good	4	32	6	10.8

*n* = 555

## Relation between BP and other variables

### Demography and parental history:

There was a significant association of BP with age (*r* = 0.16, *P* = 0.001 for SBP; *r* = 0.132, *P* = 0.012 for DBP). Overall, mean SBP and DBP were 117.17 mmHg (range 96-150) and 78.12 (range 52-100), respectively. Mean BP of the cohort increased significantly with age. This rising trend was observed in both genders but was significant only among males (*r* = 0.26, *P* = 0.000 for SBP and *r* = 0.251, *P* = 0.01 for DBP). Mean SBP among males was 118.44 (96-150), and mean DBP was 79.25 mmHg (60-96). Females had mean SBP and DBP of 115.76 (100-142) and 76.89 (52-100), respectively. The difference in mean BP among the genders was significant (*P* = 0.003 and 0.01 for SBP and DBP).

In the cohort, none were eligible to be in social class I. Mean SBP among social classes II, III, IV and V were 120.18, 120.27, 116.78 and 113.0 mmHg, respectively. The respective DBP were 81, 79.95, 77.88 and 74.44 mmHg. The incidence of hypertension was 36.6/1000 in social class III and 40/1000 in social class IV. No new cases were found in social classes II and V. This difference among the social classes was not significant.

Persons with history of parental hypertension showed significant elevation in both mean SBP (119.92 mmHg) and DBP (80.36 mmHg) compared to 116.69 mmHg and 77.73 mmHg in those without such history (*P* = 0.011). Incidence of hypertension was 50/1000 among subjects with both paternal and maternal hypertension, and 21.2 and 17.5/1000, respectively, among those with either maternal or paternal history (*P* = 0.04).

### Anthropometry:

There was a significant increase in mean SBP and mean DBP with increasing weight (*r* = 0.466 and 0.409) and height (*r* = 0.211 and 0.184). Again, a significant correlation was found between BP and BMI (*r* = 0.399 for SBP and 0.355 for DBP). For the categories underweight, normal, overweight and obesity, the mean SBP were 113.19, 117.41, 123.49 and 127 mmHg (*P* < 0.001), and the mean DBP were 74.77, 78.47, 82.4 and 86.70 mmHg, respectively (*P* < 0.001). Incidences of hypertension among underweight, normal and overweight persons were 2, 12.3 and 13.5/1000, respectively, which was statistically significant (*P* < 0.001). There was no significant effect of type of food intake, smoking and alcohol consumption on blood pressure among these adolescents and young adults.

### Lifestyle:

There was significant association between physical activity and BP (*P* = 0.016), with sedentary and mild physical activity merged into a single class interval. Here, physical activity denoted only the deliberate physical exercise by subjects (like playing, etc.) and not the whole range of activities performed by them. Mean SBP among mild, moderate and very good physical activity were 116.91, 118.59, 115.52 mmHg, respectively, and mean DBP were 78.21, 78.89 and 76.76 mmHg. Incidence of hypertension among mild, moderate and very good physical activity were 7, 11 and 10.8/1000, respectively. Mean salt intake was 10.48 g/day in the reference cohort compared to 10.84 g/day in the study cohort showing no significant difference. Mean salt intake among hypertensives was 14.8 g/day compared to 10.4 g in normotensives, (*P* < 0.001). Mean SBP among those who had an intake of < 10 g/day was 113.2 mmHg compared to 119.5 mmHg in those who had an intake of >10 g/day (*P* = 0.012). Corresponding DBP values were 75.3 mmHg and 80.43 mmHg, respectively (*P* = 0.001). Mean salt intake in persons <5^th^ percentile was 8.05 g compared to 15.51 g in >95^th^ percentile (*P* < 0.05). Incidences of HT were 2 and 16.5/1000, respectively, which was statistically significant (*P* = 0.002).

### Tracking model analysis:

Further analysis was done by clubbing the age into three groups (19-20, 21-22 and 23-24 years). The ‘Z’ scores were calculated for SBP and DBP gender-wise for the age groups [Table 4]. The baseline values for salt intake, BMI, physical activity and family history of hypertension were included in multiple regression models - with the BP values of the reference cohort of 2002 as independent variable and BP values of study cohort of 2006 as dependent variable. It was found that for each of the age-group ‘Z’ scores for BP of reference cohort was significantly predicting the BP of study cohort. However, BMI was found to significantly predict the dependent BP (both SBP and DBP) only for the age group 17-18 years, among females only. Similarly, salt intake was found to be a significant predictor only for the dependent DBP in males in 17-18 years group [Table 5].

**Table 4:** Descriptive statistics of blood pressure among study subjects

Age (years)	Gender	Mean 2002 SBP (SD)	Mean 2002 DBP (SD)	Mean 2006 SBP (SD)	Mean 2006 DBP (SD)
15-16	M	112.77(09.20)	73.46 (9.20)	114.85 (7.50)	76.24 (7.2)
17-18	M	113.50(09.50)	74.47 (9.80)	116.81 (8.20)	77.37 (7.3)
19-20	M	119.34 (12.00)	78.13(9.80)	121.95(9.90)	81.91 (7.7)
15-16	F	112.38(08.12)	74.60 (8.26)	115.31 (7.83)	76.70 (7.8)
17-18	F	113.40(10.50)	73.76 (8.30)	115.96(9.60)	77.47(7.1)
19-20	F	115.10(09.70)	76.00 (8.70)	118.30(8.50)	79.50 (6.9)

**Table 5:** Multiple regression models for blood pressure tracking

Age	Sex	Constant		2002 BP (Z score)		2002 salt		2006 BMI		2002 physical activity*		Family history†	
							
		*k*	*p*	β	*p*	β	*p*	β	*p*	β	*p*	β	*p*
Systolic blood pressure													
15-16	M	0.27	0.5	0.86	0.00	−0.02	0.7	−0.06	0.25	0.04	0.4	−0.008	0.87
17-18	M	1.01	0.03	0.85	0.00	−0.037	0.4	0.064	0.22	0.04	0.4	−0.05	0.35
19-20	M	−0.47	0.2	0.88	0.00	0.006	0.9	0.07	0.19	−0.03	0.62	0.06	0.25
15-16	F	−0.19	0.7	0.52	0.00	0.053	0.5	−0.08	0.34	0.13	0.12	−0.11	0.89
17-18	F	−1.28	0.02	0.7	0.00	0.02	0.7	0.177	0.01	0.01	0.88	0.03	0.7
19-20	F	−0.57	0.3	0.7	0.00	−0.006	0.9	0.085	0.31	−0.12	0.18	0.06	0.47
Diastolic blood pressure													
15-16	M	0.69	0.2	0.7	0.00	0.025	0.7	−0.1	0.16	−0.04	0.57	−0.14	0.89
17-18	M	0.70	0.2	0.77	0.00	0.12	0.05	−0.07	0.23	−0.02	0.71	−0.05	0.43
19-20	M	−0.25	0.6	0.83	0.00	−0.56	0.58	0.48	0.6	−0.006	0.72	0.07	0.27
15-16	F	−0.61	0.3	0.78	0.00	−0.035	0.58	−0.019	0.7	0.03	0.72	0.04	0.38
17-18	F	−1.6	0.01	0.68	0.00	−0.045	0.54	0.184	0.02	0.06	0.45	0.08	0.27
19-20	F	0.49	0.4	0.73	0.00	−0.028	0.72	−0.48	0.63	−0.93	0.35	−0.015	0.87

* Severe exercise - 4, moderate exercise - 3, mild exercise - 2, sedentary - 1;

† No family history - 1, maternal history - 2, paternal history - 3, both parents - 4 (physical activity and family history are coded as above since they are ordinal variables)

## Discussion

This study comments on the tracking of BP in a cohort representative of urban slum dwellers of Puducherry. Several studies have highlighted the importance of tracking BP. In the present study, more than 70% of those who were hypertensives in the reference cohort (>95^th^ percentile) continued in the same range, which is similar to a Spanish study,(10) which reported that more than 70% children in the upper quartile of SBP at any previous examination remained so. The Shimane heart study(11) showed 43.5% boys and 59.1% girls in cohort 1, and 25.0% boys and 56.5% girls in cohort 2 remained in extreme quintiles.

The incidence of HT in adolescents and young adults in the present study was 0.98%. The Framingham(12) study showed an incidence of 3.3% among men aged 30-39, and Menghett(13) *et al*. showed a high incidence of 6.5% among 293 children in the age group of 11-14. In the present study on adolescent and young adults, a significant increasing trend of BP was seen only among males. This is similar to the Turkish(14) and Zambian(15) studies on school children showing rise of BP with age. The latter study showed a significantly elevated mean SBP and DBP among males than females. This is comparable to other studies on populations of 13-18 years,(16) 15-24 years(17) and 15-25 years.(18) Although the present study did not find a significant association of mean BP with social class, the findings in a South Indian community(19) showed such significance.

There was a gradient of significant association of BP and HT with physical activity in the present study on adolescents as also observed by Pittsburgh study.(16) Physical fitness appears to be a graded, independent, long-term predictor of mortality from cardiovascular causes in healthy, middle-aged men.(21)

The present study found significant rise in both SBP and DBP with increasing BMI in both genders, comparable with the findings from adolescents 17 years in Jerusalem,(20) whereas another study(16) reported weight-dependent rise in BP only among males with respect to SBP alone. Several studies reported the association of BP with both weight and height.(16,21–23) Prevalence of HT was 0.5 to 3 times higher among the overweight.(24) Taiwan study(25) which followed 7685 males for over 30 years recorded an increase in incidence of HT with increasing ponderosity. The Framingham study(12) showed increased prevalence of obesity in subjects with HT as well increase in BP in established obesity. Similar findings were reported among adolescent populations in India,(26) Hungary(27) and France.(28) Such association in early childhood with SBP alone was reported by Minneapolis children's BP study(29) and British cohort.(30) Pittsburgh(16) study reported similar association only in SBP among males. Other studies on populations of 4-18 years((14) and 7-16 years((15) also identified weight as a major determinant of BP. Childhood weight gain was positively associated with adult BP.(31) Another study(13) showed that elevated BMI in childhood predicted risk of hypertension in young adulthood.

Subjects with history of parental hypertension had higher BP and higher incidence of hypertension. Zimbabwean study(34) showed that parental history before age 60 was related to offspring's hypertension. This relationship was stronger when compared to both parental histories versus none similar to the present study. Another Zimbabwean(35) study showed parental history of hypertension influenced both resting and reactivity BP.

In this study, BP was significantly predicted by baseline salt intake only in 17-18 years group in males. There is evidence that high salt intake increases BP.(32) Low sodium was reported to lower BP.(33) Likewise there was a significant predictability of BP by baseline BMI only in 17-18 years in both genders.

## Limitations

1. Since the cohort follow-up was done after 4 years, the annual incidence of hypertension was calculated from the 4 years incidence, assuming uniform yearly incidence. About 201 (26.3%) subjects (out of the 2002 cohort of 756 subjects) could not be approached even after 4 visits. Although the age composition of this 201 was comparable with the 555 of the present cohort, it is still possible that this loss to follow-up might have some effect on the results depending on the distribution of other variables.
2. The major proportion of baseline 2002 cohort consisted of adolescents (15-19 years) and some young adults (20 years). The follow up 2006 cohort consisted of lesser proportion of adolescents (19 years) and major proportion of young adults (20-24 years). The definition of hypertension differs for adolescents (>95^th^ percentile) and for adults (SBP > 140, DBP > 90). The incidence of HT was calculated individually for adolescents/young adults as two different groups and the combined incidence for the whole group was taken as addition of these two incidences.
3. Likewise, any differential drop outs with respect to age, BMI, social class, alcohol intake, smoking, physical activity and family history of hypertension might have further limitations in interpretation of results; these aspects are specifically addressed in a paper from a subsequent study.
4. In addition measurement of physical activity which included only exercise might have limited accuracy but was used to ensure comparability with the earlier cohort. Similarly the per capita salt intake was calculated as an average of the family intake since a specific dietary measurement was not within the scope of this study.

## Conclusion

Tracking is a very useful tool in early diagnosis of pre-hypertension and hypertension even among adolescents/young adults (19-24 years). The overall mean SBP and DBP were 117.17 mm Hg (range 96-150) and 78.12 (range 52-100), respectively, and annual incidence of hypertension was 9.8/1000. Baseline BP (both SBP and DBP) was the significant independent predictor of BP. Other variables like BMI and salt intake were significant predictors of BP only in a section of the study population. Hence, pertaining to adolescents/young adults, these may be termed as early risk factors.
